# Atligator Web: A Graphical User Interface for Analysis and Design of Protein–Peptide Interactions

**DOI:** 10.34133/bdr.0011

**Published:** 2023-05-04

**Authors:** Josef Paul Kynast, Birte Höcker

**Affiliations:** Department of Biochemistry, University of Bayreuth, Bayreuth, Germany.

## Abstract

A key functionality of proteins is based on their ability to form interactions with other proteins or peptides. These interactions are neither easily described nor fully understood, which is why the design of specific protein–protein interaction interfaces remains a challenge for protein engineering. We recently developed the software ATLIGATOR to extract common interaction patterns between different types of amino acids and store them in a database. The tool enables the user to better understand frequent interaction patterns and find groups of interactions. Furthermore, frequent motifs can be directly transferred from the database to a user-defined scaffold as a starting point for the engineering of new binding capabilities. Since three-dimensional visualization is a crucial part of ATLIGATOR, we created ATLIGATOR web—a web server offering an intuitive graphical user interface (GUI) available at https://atligator.uni-bayreuth.de. This new interface empowers users to apply ATLIGATOR by providing easy access with having all parts directly connected. Moreover, we extended the web by a design functionality so that, overall, ATLIGATOR web facilitates the use of ATLIGATOR with a more intuitive UI and advanced design options.

## Introduction

The specific recognition of binding partners in protein–protein or protein–peptide interactions is established by mutual interactions of amino acid residues. While each residue’s contribution shapes the binding affinity and specificity to agonist or antagonist binders, certain residue–residue interactions are more crucial than others. With this in mind, it is likely that in the context of optimized binding partners, pairwise interactions with a higher influence will be found more often than others. Following this hypothesis, Singh and Thornton [1] already identified some frequent interaction residue pairs and defined their spatial arrangement. Amino acid pairs have also been investigated energetically [2–4] or in a generalized form with focus on functional groups [5]. Within densely packed interaction surfaces, however, a pairwise residue–residue interaction is affected by its context. This structural context and identities of neighboring residues were successfully incorporated in recent analyses [6–9].

Another idea is to investigate groups of residues that act as a binding partner for single residues. For this approach, we created the software package ATLIGATOR [10]. It extracts pairwise interactions from protein structures to find patterns in frequent residue–residue pairs and stores these in a data structure called *atlas*. By varying the input structures, the structural and evolutionary context of underlying data can be modulated and compared. Moreover, by mining the *atlas* for frequent interaction groups, interaction motifs can be extracted, visualized, and analyzed. Additionally, ATLIGATOR enables direct grafting of frequent interaction motifs (*pockets*) onto a user-defined scaffold protein. Since working with three-dimensional interaction motifs or designing binding pockets is a highly visually demanding task, it also allows to plot *atlas* or *pocket* data.

To enable everyone to try and use ATLIGATOR instantly without installation, we developed a web interface. This graphical interface connects all data structures visually and even extends the design options of ATLIGATOR. It is freely accessible at https://atligator.uni-bayreuth.de and creates an intuitive starting point for working with the software.

## Results and Discussion

ATLIGATOR web expands the functionality of the ATLIGATOR python package by a user-friendly interface, thereby providing easy access and supporting users to utilize its features instantly. For the analysis of pairwise interactions, we provide a list of pre-generated *atlases* and *pocket collections* that can be explored in detail. All data can be visualized similarly to the python API. However, a unique aspect of the web interface is the connection between the different sections, i.e., an *atlas* to its input structures, *pockets* to its underlying *atlas*, etc. This connection is accomplished mainly by hyperlinks to superior data structures. Additionally, the representation of the plotted data points clearly highlights these connections in several ways: The original structural environment of data points is directly displayed by clicking on data points and, in this visualization, all atoms are labeled with their origin.

While the focus of ATLIGATOR web is the representation of data structures in a more intuitive way, we additionally provide the opportunity to design protein interaction sites. This is useful, because ATLIGATOR data can comprise potential starting points for shaping binding surfaces. Hence, by supplying a protein–protein or protein–peptide complex and defining ligand and binder chains, *pockets* can be grafted directly onto the binder chain. Additionally, manual mutations can be applied to extend or fine-tune grafts with new mutations.

### Analysis of binding interfaces

Below, we will elaborate the web interface and each section in more detail to showcase potential usage and highlight special features. To do so, we will guide through the data structures of ATLIGATOR web with the same example as examined before [10]. Namely, we will look for interaction motifs to use in a designed armadillo repeat protein to change specificity from an arginine to a leucine as a residue of the native peptide binding partner [11,12].

#### The interface

**Fig. 1.**
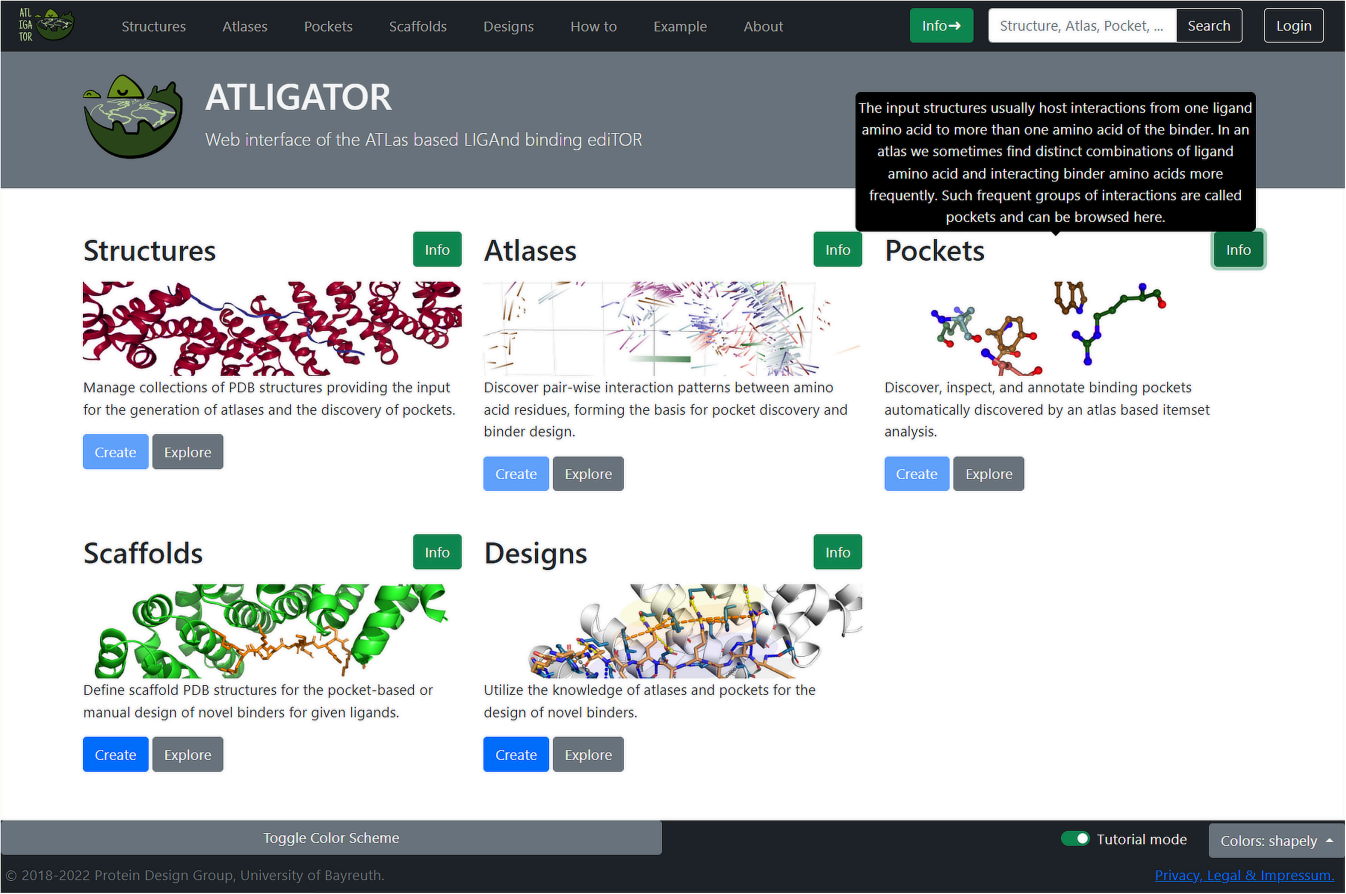
Landing page of ATLIGATOR web. It comprises links to the five main sections, namely, *Structures, Atlases, Pockets, Scaffolds,* and *Designs*. The header includes a navigation bar as well as a search box and a context box for user accounts. The footer features a switch for tutorial mode that enables one to switch on info boxes with explanations to guide new users as shown. The common color scheme can be selected and an overview of the color scheme can also be activated in the footer.

The ATLIGATOR web interface is built around five main sections, namely, *Structures*, *Atlases*, *Pockets*, *Scaffolds*, and *Designs* (see Fig. 1). The landing page gives access to all sections as well as frequently asked questions (FAQ) and an example page. The footer of each web page includes the current color scheme and a switch to activate the tutorial mode. If activated, info boxes that explain function and handling of applications are shown. We could start our redesign example in the section *Atlases*, but to understand where the *atlas* data originate, we will visit the section *Structures* first.

#### Structure collections

Interaction data stored in *atlases* or *pockets* originate from protein structures. In contrast to the ATLIGATOR python package, ATLIGATOR web integrates these structures as an explorable section. Within this section, input structures are grouped in *collections* that typically consist of all structures assigned to a SCOPe database identifier. Our example protein scaffold [Protein Data Bank (PDB) identifier 5AEI] is not classified in the SCOPe database (version 2.08) because of its synthetic nature. However, since it originates from natural armadillo repeat proteins (a.118.1.1 in SCOPe), we can utilize the structures attributed to a.118 (α-α-superhelix). The *structure collection* comprises 2,584 structures, each hosting a binder chain and all potentially interacting ligand chains. Each three-dimensional structure can be observed and downloaded individually.

#### Atlases

**Fig. 2.**
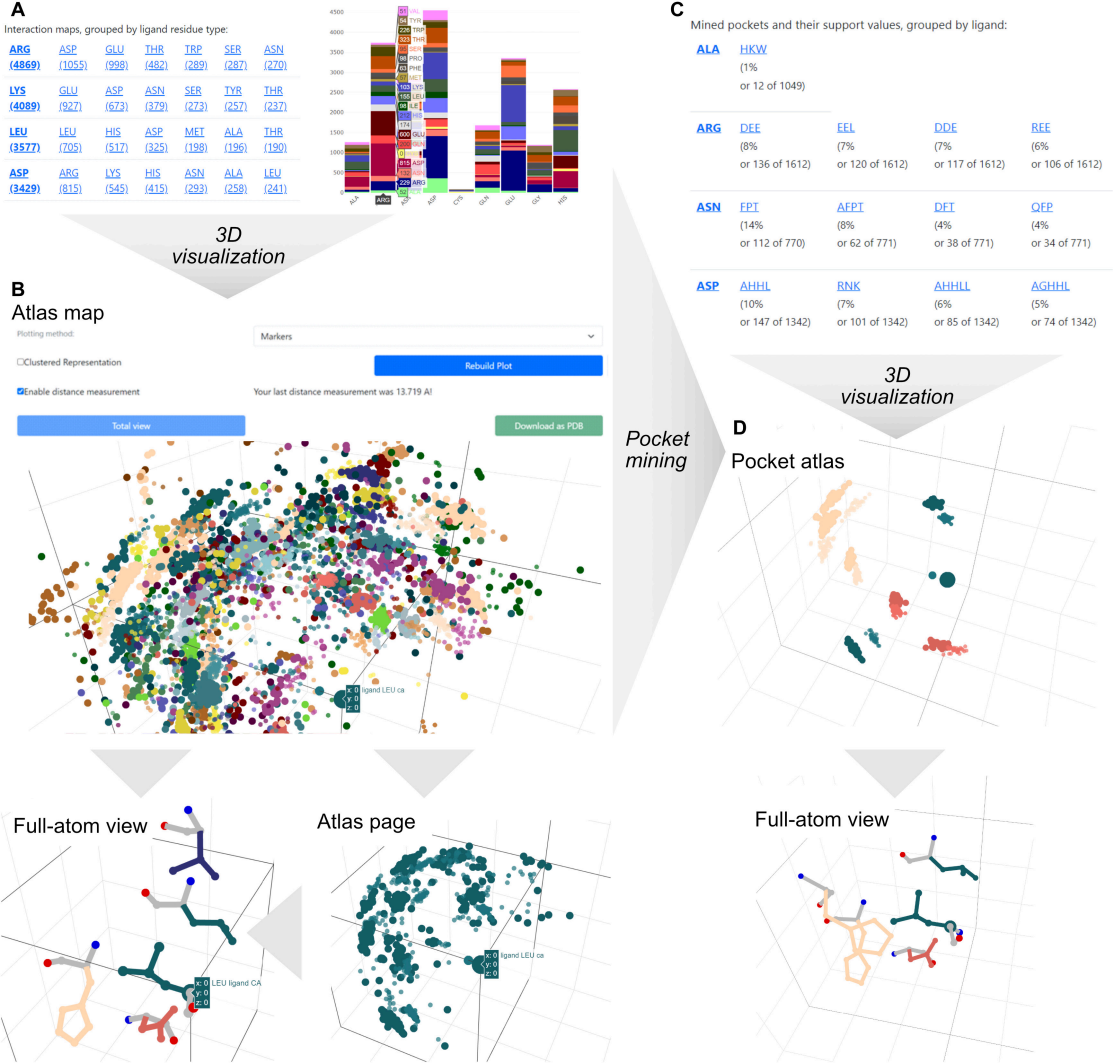
Visualization of *atlas* and *pockets*. Starting at an *atlas*, the ATLIGATOR web gives an overview of the *atlas’* content (A), and further offers the option to plot and visualize its content in three dimensions (B). *Atlas maps* and *pages* can be inspected as Cα–Cβ plots as well as in a full-atom view while concentrating on just one ligand origin. *Pockets* derived from mining the *atlas* are grouped in *pocket collections.* In addition to an overview of the contained pockets (C), all pockets can be plotted in Cα–Cβ plots as well as in full-atom view (D).

*Atlases* are based on pairwise interactions derived from single *structure collections*. Thus, *atlases* in the web interface contain a link to the corresponding *structure collection*. As a landing page, *atlas* statistics visualize the frequency of the data points. The a.118-based *atlas* contains 43,645 data points, i.e., pairwise interactions. Our design target amino acid leucine (Leu) comprises 3,577 interactions—with other Leu (705), histidine (His; 517), and aspartate (Asp; 325) being the most frequent binding partners (Fig. 2A). The *atlas* can be browsed for *atlas* maps and *atlas* pages where the number of data points is described and the data points can be viewed in three-dimensional plots. The plot of leucine’s *atlas map* reveals point clouds with high density of those Leu/His/Asp interactions that can be revisited, e.g., for the leucine-to-leucine interactions in the corresponding *atlas* page (Fig. 2B). Clicking on individual data points exhibits the entire side chains of both interaction partners of the underlying pair (Fig. 2B—full-atom view). Furthermore, pairwise distances of all displayed atoms can be measured. The positions in combination with their Cα to Cβ orientation can already provide first ideas for the design of such interactions.

#### Pockets

Frequent groups of interactions found in an *atlas* that result from mining all atlas data points for recurring motifs are defined as *pockets* [10]*.* On the web interface, *pockets* from the same *atlas* are grouped in *pocket collections*. After opening the a.118 *pocket collection*, statistics are displayed for all ligand amino acid types (Fig. 2C) of which a DHL *pocket* was the most abundant Leu pocket. This *pocket*, being composed of the three amino acids Asp (D), His (H), and Leu (L), was observed in interaction with 10% of all Leu ligand residues of the initial *atlas*.

The *pocket* and its data points are visualized in different representations: First, the most representative pocket, which corresponds to the pocket instance with the lowest deviation from the item set clusters’ centroids [10], is visualized in a full-atom stick representation. Second, the clustered data points (centroids of clusters) and all data points of the pocket *atlas* are initially shown in *atlas* representation with ligand and binder Cα and Cβ atoms as bubbles (Fig. 2D). The pocket *atlas* contains all data points and represents a filtered instance of the original *atlas.* It exposes three spatially restricted clusters for His Cα positions as well as two clusters for each Leu and Asp (see Fig. 2D) and provides two additional types of representations. On the one hand, all ligand residue atoms can be displayed separately to see the distribution of ligand side chain conformations. On the other hand, after clicking on a ligand or binder atom, the corresponding single pocket instance is plotted with all residues in full-atom representation (Fig. 2D). The full-atom representation of our example reveals two interaction motifs: First, Leu being trapped between 2 His, 1 Asp, and 1 Leu, where the Leu–Leu interaction is dominated by side chain–backbone interactions. Second, Leu is attacked by Leu and His roughly at a 90° angle with the Asp facing its carboxy group to the backbone of the target Leu. These single *pocket* instances provide promising starting points for our redesign and can be downloaded as .pdb files or used directly for grafting onto a user-defined scaffold.

### Redesign of binding interfaces

While the analysis of frequent motifs helps to extract ideas for the redesign of interaction interfaces, we additionally included design tools to directly use this information on specific binding interfaces. Those tools will be briefly introduced below.

#### Scaffolds

Proteins on which ATLIGATOR *pockets* shall be grafted are called scaffolds. Users can upload their own protein structures, hosting two or more polypeptide chains. One of these chains has to be defined as the ligand chain, comprising the residue on which basis the pocket should be selected. The chain has to be defined as the binder chain that is mutated eventually. Afterwards, mutable positions must be selected to finalize the scaffold preparation.

#### Designs

**Fig. 3.**
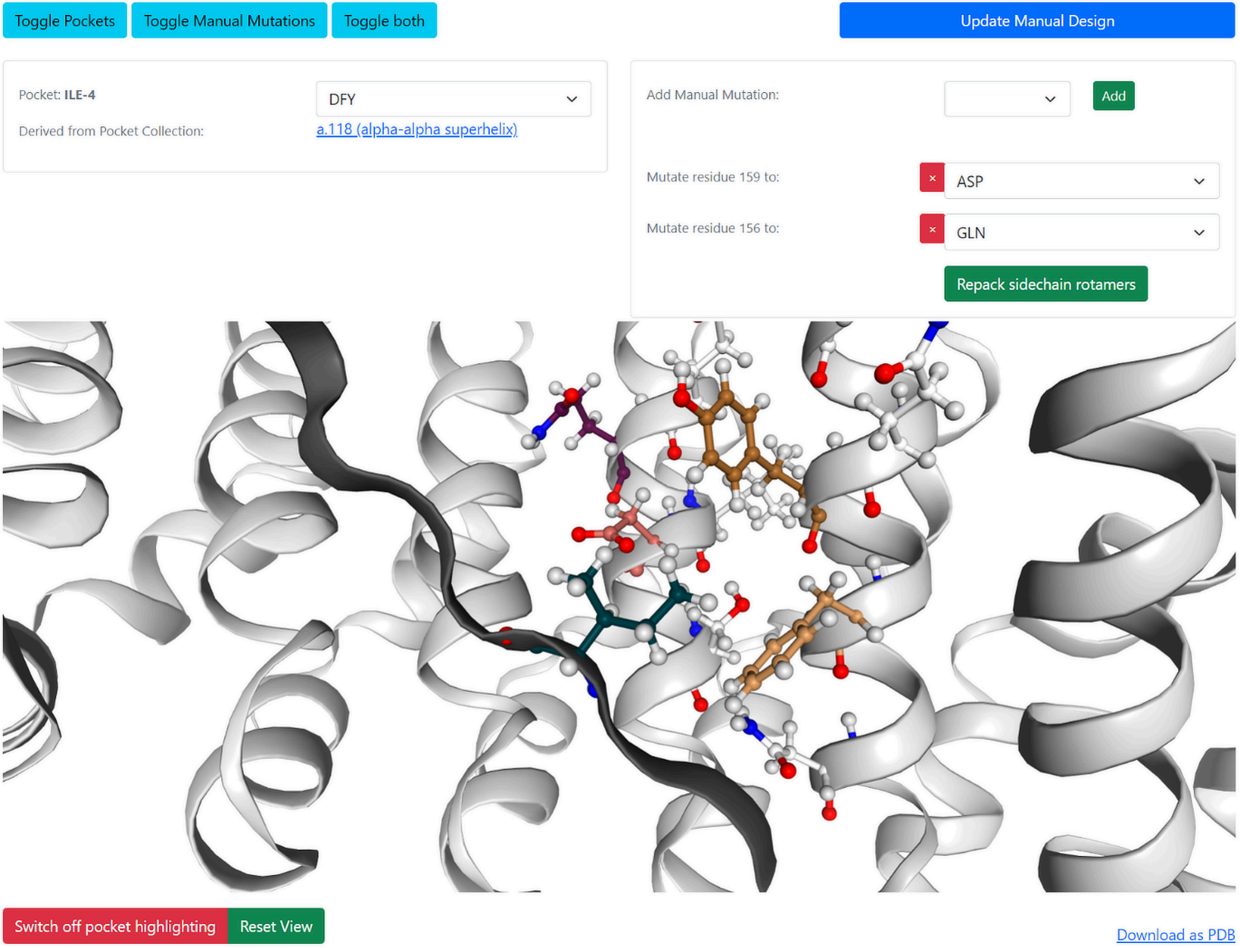
Design interface including grafting and manual mutations. Two tiles provide dropdown menus to design the scaffold protein by grafting a *pocket* of choice or applying manual mutations. Changes are applied after clicking the “Update Manual Design” button or the “Repack sidechain rotamers” button, while the latter also applies the Rosetta repacking protocol to the mutable side chain rotamers. Changes can be observed directly in the preview tile with an option to colorfully highlight the mutated positions or by downloading the .pdb file of the designed protein.

A design is defined by the scaffold and the residue types that will be used in mutable ligand positions. Each design can harbor multiple design tasks where the binder can be altered by applying different mutations. After selecting a pocket collection for the design task, the user can start designing. Within the tool *manual graft*, two ways of mutating the scaffold are implemented. The first method is based on choosing a pocket from the pocket collection for each ligand residue that will be grafted automatically onto the scaffold (Fig. 3). The grafting applies mutations based on the selected pocket onto the best fitting mutable binder positions as in ATLIGATOR [10]. However, the selection of mutated positions heavily depends on the underlying pocket data, which might not fit flawlessly with the scaffold if the geometry of the interaction is not perfectly alignable. Moreover, the best graft is not guaranteed to fit better than the next best grafts due to the lack of scoring for resulting shape complementarity or actual interactions. Thus, as the second mutation method, we implemented an option to choose manual mutations. Mutations can be added independently for all mutable binder residues and override mutations from pocket grafting.

Since grafted mutations incorporate the side chain conformer of the pocket data point and the manual mutations do not include a side chain conformer at all, the rotamers do not resemble realistic conformations. Thus, a refinement of the designed interface might be necessary to judge the design’s quality. Manual graft offers to repack the mutable interface using Rosetta’s fixbb protocol to generate more realistic side chain conformations [13].

## Conclusion

The development of ATLIGATOR opens up the possibility to collect and learn from protein–protein interactions in a streamlined and automated fashion. ATLIGATOR web empowers users to leverage its functionality in a user-friendly and easily accessible environment. Additionally, the web interface extends ATLIGATOR tools with novel functions like connecting *atlas* data points to the underlying structural data and an advanced design tool for pocket grafting and rational design. In summary, the easy access via this graphical interface enables a broader user base to apply ATLIGATOR and helps to understand its principles. Thus, ATLIGATOR web might also be a starting point and encourage users to directly apply the python package for extended analysis and design.

## Implementation

### Code implementation

The web interface for ATLIGATOR is implemented in the Python 3 web framework Django [14] in combination with uWSGI as a reverse proxy and javascript for client code handling. We use jQuery and bootstrap 5 as css and javascript frameworks for web design and celery in combination with rabbitMQ for asynchronous task handling.

### Visualization

The JavaScript library plotly.js is used for responsive plotting of statistics data. Three-dimensional plotting is performed by two JavaScript libraries. Plotly.js is handling *atlas* and *pockets* as well as full atom plotting of these [15]. NGL viewer is implemented for visualization of proteins and protein complexes [16].

### Availability

The sections *structures*, *atlases*, and *pockets* are openly accessible for discovering the data in the pre-built data structures. The protein structures were collected by searching the SCOPe database [17] with the corresponding queries. The starting *structure collections* were chosen based on known peptide binding capabilities, a diverse classification in SCOPe and multitude of available structures in the PDB. The protein structure files were preprocessed to generate files with one protein chain and cropped ligand chains. The resulting structure files were used for *atlas* generation and subsequent *pocket* mining. All processing parameters were used as previously described [10]. The design sections including *scaffolds* and *designs* can be fully explored via a user account that only requires a username, email address, and password. We also offer a scaffold and design example that correspond to the ones discussed above.

### Documentation

The web server includes a tutorial mode that offers comments and explanations for the different features and sections directly on the web pages. It also links to videos for showcasing sections of ATLIGATOR web to enable an easier start.

## Data Availability

ATLIGATOR web is available at https://atligator.uni-bayreuth.de. It is based on the ATLIGATOR code, which is available at https://github.com/Hoecker-Lab/atligator.

## References

[1] Singh J, Thornton JM. *Atlas of protein side-chain interactions*. Oxford (UK): IRL Press at Oxford University Press; 1992.

[2] Berka K, Laskowski RA, Hobza P, Vondrášek J. Energy matrix of structurally important side-chain/side-chain interactions in proteins. J Chem Theor Comput. 2010;6(7):2191–2203. 10.1021/ct100007y 26615945

[3] Berka K, Laskowski R, Riley KE, Hobza P, Vondrášek J. Representative amino acid side chain interactions in proteins. A comparison of highly accurate correlated ab initio quantum chemical and empirical potential procedures. J Chem Theor Comput. 2009;5(4):982–992. 10.1021/ct800508v 26609607

[4] Galgonek J, Vymětal J, Jakubec D, Vondrášek J. Amino acid interaction (INTAA) web server. Nucleic Acids Res. 2017;45(W1):W388–W392. 28472475 10.1093/nar/gkx352 PMC5570164

[5] Polizzi NF, Degrado WF. A defined structural unit enables de novo design of small-molecule-binding proteins. Science. 2020;369(6508):1227–1233. 32883865 10.1126/science.abb8330 PMC7526616

[6] Liu S, Xiang X, Gao X, Liu H. Neighborhood preference of amino acids in protein structures and its applications in protein structure assessment. Sci Rep. 2020;10(1): Article 4371. 32152349 10.1038/s41598-020-61205-w PMC7062742

[7] MacKenzie CO, Zhou J, Grigoryan G. Tertiary alphabet for the observable protein structural universe. Proc Natl Acad Sci USA. 2016;113(47):E7438–E7447. 27810958 10.1073/pnas.1607178113 PMC5127300

[8] Swanson S, Sivaraman V, Grigoryan G, Keating AE. Tertiary motifs as building blocks for the design of protein-binding peptides. Protein Sci. 2022;31(6): Article e4322. 35634780 10.1002/pro.4322 PMC9088223

[9] Zhou J, Panaitiu AE, Grigoryan G. A general-purpose protein design framework based on mining sequence–structure relationships in known protein structures. Proc Natl Acad Sci USA. 2019;117(2):1059–1068. 31892539 10.1073/pnas.1908723117 PMC6969538

[10] Kynast JP, Schwägerl F, Höcker B. ATLIGATOR: Editing protein interactions with an atlas-based approach. Bioinformatics. 2022;38(23):5199–5205. 36259946 10.1093/bioinformatics/btac685 PMC9710554

[11] Gisdon FJ, Kynast JP, Ayyildiz M, Hine AV, Plückthun A, Höcker B. Modular peptide binders—Development of a predictive technology as alternative for reagent antibodies. Biol Chem. 2010;403(5–6):535–543. 10.1515/hsz-2021-0384 35089661

[12] Hansen S, Tremmel D, Madhurantakam C, Reichen C, Mittl PRE, Plückthun A. Structure and energetic contributions of a designed modular peptide-binding protein with picomolar affinity. J Am Chem Soc. 2016;138(10):3526–3532. 26878586 10.1021/jacs.6b00099

[13] Kuhlman B, Baker D. Native protein sequences are close to optimal for their structures. Proc Natl Acad Sci. 2000;97(19): 10383–10388. 10984534 10.1073/pnas.97.19.10383 PMC27033

[14] Django. Django (Version 4.0). Lawrence (KS): Django Software Foundation; 2022. https://www.djangoproject.com/.

[15] Plotly Technologies Inc. Collaborative data science. Montreal (Canada): Plotly Technologies Inc.; 2015.

[16] Rose AS, Hildebrand PW. NGL viewer: A web application for molecular visualization. Nucleic Acids Res. 2015;43(W1):W576–W579. 25925569 10.1093/nar/gkv402 PMC4489237

[17] Fox NK, Brenner SE, Chandonia J-M. SCOPe: Structural classification of proteins—Extended, integrating SCOP and ASTRAL data and classification of new structures. Nucleic Acids Res. 2014;42(Database issue):D304–D309. 24304899 10.1093/nar/gkt1240 PMC3965108

